# The Strength Model of Self-Control in Sport and Exercise Psychology

**DOI:** 10.3389/fpsyg.2016.00314

**Published:** 2016-03-02

**Authors:** Chris Englert

**Affiliations:** Department of Educational Psychology, Institute of Educational Science, University of BernBern, Switzerland

**Keywords:** ego depletion, emotion, self-control, self-regulation, sport, willpower

## Abstract

The strength model of self-control assumes that all acts of self-control (e.g., emotion regulation, persistence) are empowered by a single global metaphorical strength that has limited capacity. This strength can become temporarily depleted after a primary self-control act, which, in turn, can impair performance in subsequent acts of self-control. Recently, the assumptions of the strength model of self-control also have been adopted and tested in the field of sport and exercise psychology. The present review paper aims to give an overview of recent developments in self-control research based on the strength model of self-control. Furthermore, recent research on interventions on how to improve and revitalize self-control strength will be presented. Finally, the strength model of self-control has been criticized lately, as well as expanded in scope, so the present paper will also discuss alternative explanations of why previous acts of self-control can lead to impaired performance in sport and exercise.

In sports, for top-level performance, it is highly important to control one’s impulses or behavioral tendencies: for instance, athletes need to downregulate their anxiety levels in high-pressure contexts (e.g., sporting competitions) in order to get calmer and more focused on the actual task at hand (e.g., performing a basketball free-throw; e.g., Hill et al., 2010), force themselves to work persistently on a straining physical exercise (e.g., Wagstaff, 2014), or force themselves to adhere to work out plans over extended time periods (e.g., Bandura, 2005). However, self-control does not always work. One explanation for lapses in self-control behavior is given by the strength model of self-control (e.g., Baumeister et al., 1994, 1998). According to Baumeister et al. (1994, 1998), a self-control act can be described as a process by which an individual tries to volitionally control or override dominant behaviors or response tendencies in order to achieve a specific goal. In regard to the examples given above, the predominant/automatic response tendency in high pressure contexts would be to experience high arousal and anxiety, to prematurely quit straining physical tasks, or to relax instead of following a tight workout schedule. All acts of self-control are based on one global energy resource (e.g., Baumeister et al., 2007). However, this resource, or strength, is assumed to have a limited capacity, and there are inter-individual dispositional differences in this capacity (i.e., trait self-control strength; Tangney et al., 2004). Individuals also differ in the amount of momentarily available self-control strength (i.e., state self-control strength; Tangney et al., 2004). After having worked on a self-control task, the strength can become temporarily depleted and does not immediately replenish: This state of temporary self-control exhaustion is termed *ego depletion* (e.g., Baumeister et al., 1994). The fact that there is carry-over effect of a primary self-control act on a secondary self-control act is also the main difference between the strength model of self-control and for instance models of cognitive load (e.g., Sweller, 1988). According to cognitive load theory, performing two tasks at the same time impairs performance while the strength model proposes that performance impairments occur in a second task being performed *after* the primary task. In a state of ego depletion, subsequent self-control performance can be impaired. For instance, in laboratory settings, ego depleted participants were less persistent in an anagram task (e.g., Muraven et al., 1998), had troubles regulating their emotions while watching an emotionally arousing video clip (e.g., Schmeichel, 2007), or made more mistakes and displayed longer response latencies in the Stroop task (Stroop, 1935), which can be considered as a classic example of a self-control task (e.g., Richeson and Shelton, 2003).

Hagger et al. (2010a) published a review on ego depletion in sports and exercise behavior. Therefore, to avoid redundancy, the aim of the present paper is to extend their review and to report recent developments in ego depletion research since Hagger and colleagues’ publication. Additionally, this paper aims at discussing research questions regarding the validity of the strength model of self-control which have recently opened up and to introduce alternative theoretical models to explain impaired self-control performance following a primary self-control act. The structure of this review paper is as follows: First, the two-task paradigm—the primary experimental approach in ego depletion research—will be introduced. Then, recent findings from ego depletion research in sport and exercise psychology will be presented. Another section will focus on possible interventions on how to improve and revitalize self-control strength. Finally, recent developments and extensions of the strength model of self-control will be discussed in more detail.

## The Two-Task Paradigm

To test the assumptions of the strength model of self-control, researchers are mostly applying the so-called two-task paradigm (e.g., Baumeister et al., 1994). Within this experimental approach, participants are randomly assigned to a depletion condition or a control condition. In a first step, participants work on a similar primary task. However, in the depletion condition, it is necessary to control oneself, whereas in the control condition, self-control is not required. In a second step, participants then work on an identical task that requires the exertion of self-control strength from both conditions. Research has reliably shown significant differences in performance between the experimental conditions in the second task as participants from the depletion condition reliably perform significantly worse (e.g., Hagger et al., 2010b). Speaking in terms of the strength model of self-control, in the depletion condition, self-control strength experimentally becomes depleted after the first task, whereas it remains intact in the control condition. There is a carry-over effect on the secondary task because self-control strength does not recover directly after finishing the primary task (e.g., Baumeister et al., 2007). For instance, in a study by Muraven et al. (1998), the primary task was to watch a sad video clip. In the depletion condition, participants were instructed to suppress any kind of emotional response (i.e., a self-control act). In the control condition, participants simply watched the clip without any additional instructions. As a secondary task, participants worked on an identical secondary task, namely squeezing a handgrip. Squeezing a handgrip becomes tiring and painful after some time and one needs to exert self-control in order to force oneself to keep squeezing the handgrip (e.g., Bray et al., 2012). Results revealed that performance in the depletion condition was significantly worse than in the control condition. It is important to keep in mind that the effect of ego depletion is not domain specific, meaning that the primary self-control task may require a different form of self-control than will the secondary self-control task (e.g., Baumeister et al., 1998). For instance, a primary cognitive task can have a carry-over effect on a physical task (e.g., Bray et al., 2012). Hagger et al. (2010b) conducted a meta-analysis on 83 studies on ego depletion and found a medium-to-large effect of ego depletion on self-control performance.

## Ego Depletion and Athletic Performance

In the following section, an overview of current research in the field of sport and exercise will be given that is based on the strength model of self-control (cf., Audiffren and André, 2015). First, the role of self-control strength on athletic performance under pressure will be presented. Then, it will be shown that self-control strength is also important in sports tasks requiring high levels of persistence. In a next step, it will be discussed that it is also necessary to exert self-control strength to adhere to workout schedules over an extended period. Finally, it will be argued that self-control strength also seems critical in athletic tasks which require the overriding of predominant behavioral impulses.

### Ego Depletion and Athletic Performance under Pressure

In high-pressure contexts (e.g., sporting competitions), athletes are not always capable of performing at their highest level, meaning that they display worse performance than they are generally capable of (e.g., Beilock and Gray, 2007). Baumeister (1984) termed this phenomenon choking under pressure. The explanation for this effect is that high-pressure contexts are often associated with high levels of state anxiety (e.g., Gucciardi et al., 2010). In this context, anxiety can be understood as an aversive emotional and motivational state occurring in threatening circumstances (Eysenck et al., 2007). Eysenck et al. (2007) propose that under high levels of state anxiety individuals have an *automatic* tendency to ruminate and to worry about the quality of their performance, which may interfere with their ability to volitionally regulate their attention and to focus their attention on the physical task at hand (e.g., the relevant target areas on a dart board; e.g., Nibbeling et al., 2012; for a broader discussion on performance under pressure, see also Beilock and Carr, 2001). Schmeichel and Baumeister (2010) define selective attention regulation as an act of focusing attention on one subset of the environment while ignoring other attention-grabbing subsets of the environment. According to Eysenck et al. (2007) individuals can counteract the automatic tendency to focus on these task-irrelevant thoughts by investing additional effort or by initiating self-regulatory processes. However, the mixed results on the anxiety–performance relationship indicate that individuals are not always capable of volitionally regulating their attention: there are several studies in which high-anxiety levels led to poor athletic performance (e.g., Behan and Wilson, 2008; Wilson et al., 2009), but there are also several studies in which there was no negative anxiety–performance relationship (e.g., Craft et al., 2003; Woodman and Hardy, 2003).

The strength model of self-control (e.g., Baumeister et al., 1994) offers an explanation for these mixed results because the momentary availability of self-control strength may determine under which conditions athletes are more likely to choke under pressure (cf., Englert and Bertrams, 2015a). As previously mentioned, efficient attention regulation, which is required for top-level athletic performance in far-aiming tasks, can be impaired under high-anxiety levels (e.g., Wilson et al., 2009). According to Schmeichel and Baumeister (2010), volitionally regulating attention is a self-control act which can suffer from ego depletion. Consequently, primarily there should be a negative effect of anxiety on athletic performance in far-aiming tasks in a state of ego depletion. If self-control strength has not been depleted by a primary self-control task, efficient attention regulation should still be possible despite the high-anxiety levels. In a series of studies, Englert and Bertrams (2012) found support for this assumption because there was only a negative anxiety–performance relationship in participants with depleted self-control strength: under ego depletion, anxious participants performed worse in a dart-throwing task, scored less free-throws, and displayed impaired performance in a dexterity task (Englert and Bertrams, 2013). Furthermore, anxious individuals displayed a less efficient gaze behavior (i.e., significantly shorter final fixation durations) in a state of ego depletion as opposed to a state with temporarily available self-control strength while performing a dart throwing task, indicating that depleted individuals cannot volitionally counteract the negative anxiety effects on attention regulation (Englert et al., 2015c). Finally, a recent study revealed that anxious participants paid more attention to an irrelevant audio stream in a state of ego depletion as opposed to a state with temporarily available self-control strength while performing a basketball free-throw task (i.e., depleted participants realized that there was a voice change in the audio stream), which also supports the assumption that efficient attention regulation under pressure is primarily affected in a state of ego depletion (Englert et al., 2015a).

Taking the previous results together, ego depletion may explain the inconsistent database on the anxiety–performance relationship in sports (e.g., Craft et al., 2003; Woodman and Hardy, 2003; Behan and Wilson, 2008; Wilson et al., 2009): With temporarily available self-control strength, athletes can counteract the negative effects of anxiety on attention regulation and can consequently keep up their performance. In a state of ego depletion, however, attention regulation becomes disrupted under pressure, which leads to impaired performance in far-aiming tasks.

### Ego Depletion and Persistence in Sports

Previous laboratory research has shown that working persistently on rather unpleasant tasks requires self-control strength, for instance, depleted participants quit earlier on unsolvable anagrams (e.g., Muraven et al., 1998). This finding has also been transferred to the sport and exercise domain to explain lapses in persistence in straining physical exercises.

One task that is frequently administered to assess persistent performance is squeezing an isometric handgrip for as long as possible (e.g., Muraven et al., 1998; Ciarocco et al., 2001; Vohs et al., 2005; Finkel et al., 2006). Squeezing a handgrip can become tiring and painful after some time, which is why self-control strength needs to be invested to resist the urge to stop the task (cf., Muraven et al., 1998). An important study on ego depletion and persistence in sports has been conducted by Bray et al. (2008, 2011). In their study, participants had to squeeze an isometric handgrip as long as possible at two times of measurement. Additionally, EMG activity in the working forearm muscles was measured during both times of measurement. Between the two times of measurement, participants worked on a Stroop task (Stroop, 1935), as self-control strength was experimentally depleted in a depletion condition and remained intact in a control condition. Performance in the depletion condition significantly decreased from before to after the depletion of self-control strength, whereas there were no significant performance differences between the two times of measurement in squeezing duration for the control condition. Additionally, EMG activation significantly increased in the depletion condition compared to the control condition (cf., Graham et al., 2014b).

In a study by Dorris et al. (2012), professional athletes were instructed to perform as many press-ups (study 1) or sit-ups (study 2) as possible at two times of measurement. Prior to the second time of measurement, participants’ self-control strength was experimentally depleted by a reliable self-control task. The results revealed that athletes performed significantly fewer press-ups and significantly fewer sit-ups in a state of ego depletion. Considering that the results are based on a within-subjects design, it can be reasoned that the athletes in the respective studies should generally have been capable of performing at a similar level at both times of measurement. However, having worked on a short self-control task before performing the physical exercise negatively affected the performance output. So, even well-elaborated physical exercises can suffer from a temporary depletion of self-control strength.

Similar results have been found by Englert and Wolff (2015). In their study, participants performed an indoor cycling task at two times of measurement (one week apart): in a state of ego depletion and under neutral conditions in a counterbalanced order. The participants were instructed to cycle as fast as possible at a fixed gear for an 18-min period at both times of measurement. Hierarchical linear modeling revealed that under ego depletion, participants displayed a lower power output. Just as in the study by Dorris et al. (2012), the authors used a within-subjects design, meaning that participants should have performed at a comparable level at both times of measurement. Nevertheless, the previous exertion of self-control strength in an unrelated primary task negatively affected participants’ persistence in the straining cycling task.

Wagstaff (2014) conducted a comparable study and reported a similar pattern of results. In his study, participants had to perform a 10-km cycling time trial while maximum heart rate, power output, and perceived physical exertion were assessed. Before starting the cycling task, participants watched an upsetting video clip (cf., Schmeichel, 2007). Participants from a depletion condition had to suppress their emotional responses to this video clip, which requires the exertion of self-control strength (cf., Schmeichel, 2007). Consequently, depleted participants were slower in the cycling task, had lower mean power outputs, displayed a lower maximum heart rate, and reported greater physical exertion than participants that did not have to suppress their emotional responses to the video clip.

### Ego Depletion and Regular Physical Activity

Even though individuals often have the intention to work out, they do not always translate their intentions into actual exercise behavior (i.e., intention–behavior gap; e.g., Rhodes et al., 2008). According to Martin Ginis and Bray (2010), the ability to adhere to workout routines or exercise plans requires self-control (see also Bandura, 2005) and may thus depend on self-control strength (see also Oaten and Cheng, 2006). This means that individuals need to block out potential distractions or temptations (e.g., tempting foods) in order to achieve their long-term goals (e.g., losing weight; e.g., Vohs and Heatherton, 2000). Therefore, self-control needs to be invested in order to bridge potential hurdles that may interfere with exercise adherence (e.g., Iso-Ahola, 2013). Several researchers have criticized traditional models of physical activity (e.g., Theory of Planned Behavior; e.g., Ajzen, 1991), claiming they cannot sufficiently explain lapses in exercise adherence (e.g., Sniehotta et al., 2014; see also Rebar et al., 2015), and that other psychological constructs are needed to explain the intention–behavior gap. Hagger (2014) suggested that trait self-control strength may prove to be an important psychological variable in that regard. On a state level, a lab-based study by Martin Ginis and Bray (2010) demonstrated that ego depletion predicted exercise adherence over an 8-weeks period. However, this study did not assess actual exercise behavior.

The importance of self-control strength for physical activity is also highlighted in a study by Bertrams and Englert (2013) in which they assessed participants’ trait self-control strength and asked participants to indicate how often they intended to work out during the week, and how often they actually did work out during the week. Results revealed that higher levels of self-control strength were associated with a smaller intention–behavior gap. Just as in the study by Martin Ginis and Bray (2010), however, physical activity was not directly measured. Finally, Toering and Jordet (2015) also reported that higher levels of trait self-control strength seem to be beneficial for exercise adherence because in their study, professional soccer players reported higher levels of trait self-control strength compared to the general population. Higher trait self-control strength was also related to a more professional lifestyle, more time spent at training facilities, and less time spent on social and play activities.

Interestingly, in their review article on the strength model of self-control, Hagger et al. (2010a) mentioned the necessity to adopt the strength model to explain exercise behavior (see also Hagger et al., 2009; Hagger, 2010). They also complained that traditional models of physical activity primarily focus on intention formation, but do not explain how intentions are transformed into actual exercise behavior (Hagger et al., 2002). The authors concluded that self-control strength may be a decisive variable that enables one to transform a workout intention (e.g., going to the gym twice a week) into actual workout behavior (e.g., actually go to the gym twice a week; see also Mann et al., 2013). Although research on self-control strength and exercise adherence is rather descriptive at the moment, the studies mentioned above indicate that the strength model of self-control (e.g., Baumeister et al., 1994) could make a valuable contribution in that regard, and future research should also focus on investigating the influence of state self-control strength on the intention–behavior gap.

### Ego Depletion and Impulse Regulation in Sports

The ability to volitionally suppress and override impulses depends on the availability of self-control strength (e.g., Baumeister et al., 1998). In laboratory settings, it has been shown that under ego depletion, participants are less capable of volitionally controlling their motor impulses (e.g., Finkel et al., 2006), and perform worse on the Stroop task (e.g., Richeson and Shelton, 2003). As previously mentioned, the Stroop task (Stroop, 1935) is a classic example of a self-control task: One needs to inhibit the impulse of naming the color word and instead to name the font color of the respective word. These findings can also be adopted to explain lapses in impulse regulation in sport settings.

In a study by McEwan et al. (2013), participants were randomly assigned to a depletion or a non-depletion condition, and performed a dart-throwing task at two times of measurement: before the manipulation of self-control strength (T1), and after the manipulation of self-control strength (T2). The dart-throwing task involved impulse regulation because participants were only allowed to throw the darts when they saw a green light flash, and had to inhibit their throwing impulse when a red or yellow light flash was displayed. They were instructed to aim at the bull’s eye, and the average distance of the dart to the bull’s eye served as the measure of throwing accuracy. As expected, depleted participants were less accurate and were also less adept in controlling their impulses because they were significantly slower in initiating their throwing motion following the green light flash.

Two recent studies demonstrated that ego depletion also seems to be critical for the quality of the sprint start in track and field, which is a task that requires impulse regulation. While waiting for the starting signal in the starting block, on the one hand, an athlete has to exert self-control to inhibit the impulse of initiating the sprinting motion too soon, as a false start may lead to immediate disqualification from the competition (International Association of Athletics Federations, 2013). On the other hand, self-control is also necessary to initiate the sprinting motion as quickly as possible following the starting signal. Englert and Bertrams (2014a) assumed that ego depletion affects sprint starts differentially depending on the level of expertise of the respective athlete. According to their line of argumentation, in athletes with track and field experience, the dominant tendency while in the starting block is to make sure to avoid a false start and disqualification at any cost, meaning that self-control strength is necessary to volitionally override this tendency. Indeed, in their study, depleted experienced athletes displayed slower reaction times after the starting signal compared to a non-depleted series of sprint starts. Ego depletion did not affect the number of false starts, further supporting the idea that professional athletes want to avoid false starts.

In another study with inexperienced track and field athletes (i.e., female soccer players), the pattern of results was also as expected: Englert et al. (2015b) reasoned that for inexperienced athletes, the dominant tendency while waiting in the starting block may not be to avoid a false start because inexperienced athletes are not fully aware of the drastic consequences following a false start. Instead, the dominant tendency should be to initiate the sprinting motion as quickly as possible. Therefore, self-control strength should be required to volitionally override the impulse of starting too soon. As expected, depleted participants were less adept in controlling their motor impulses and displayed a higher number of false starts as opposed to states with available self-control strength.

Taken together, the studies mentioned above demonstrate that self-control strength helps athletes to control their impulses, which enables better athletic performance.

## Possible Interventions

The studies reviewed thus far reliably show that a temporary depletion of self-control strength can impair athletic performance on several levels. These findings highlight the necessity to identify strategies to prevent a loss of self-control strength. Baumeister et al. (1998) compared self-control strength to a human muscle. This muscle can become temporarily depleted after having exerted self-control strength. Also in line with the muscle metaphor, this resource can be strengthened and by doing such, its capacity can be increased (Baumeister et al., 2006). For instance, it has been reliably shown that exerting self-control strength regularly over a 2-weeks period led to better self-control performance in unrelated self-control tasks in the long run (e.g., Gailliot et al., 2004; Muraven, 2010). In a similar fashion, a study by Oaten and Cheng (2006) revealed that participating in regular physical exercise over a 2-months period led to better self-control performance in other unrelated self-control domains, underpinning that it requires self-control strength to adhere to a regular workout schedule.

Bray et al. (2015) tested this training approach in sports and investigated the effects of a 2-weeks self-control training on performance in a maximal incremental exercise test on a cycle ergometer. At the first time of measurement, self-control strength was experimentally depleted and participants performed the maximal incremental exercise test as a measure of baseline performance. Then, participants were randomly assigned to a training or a control condition. The self-control training consisted of squeezing a handgrip (e.g., Muraven et al., 1998) twice a day for as long as possible, each day over the 2-weeks period, while participants in the control condition did not receive any further instructions. After 2 weeks, participants reported back to the laboratory, self-control was experimentally depleted, and they again performed the maximal incremental exercise test. There was a large, significant training effect on performance in the maximal incremental exercise test because participants from the training condition performed significantly better. So, in line with the notion that self-control strength is not domain-specific (e.g., Baumeister et al., 1998), regularly exerting self-control strength in one domain (i.e., squeezing a handgrip) had a carry-over effect on exercise performance from a different domain.

Apart from improving self-control strength in the long run, there are also ways to replenish depleted self-control strength in a given situation. Relaxation techniques (e.g., mediation, autogenic training; e.g., Greenspan and Feltz, 1989) after having performed a self-control task (Tyler and Burns, 2008) or mindfulness mediation (Friese et al., 2012) have proven to be valuable approaches in that regard. Using the muscle analogy of Baumeister et al. (1998), applying relaxation techniques leads to a quicker regeneration of an exhausted muscle, in this case an exhausted/depleted self-control strength. Relaxation techniques have a long-standing tradition in sport and exercise psychology (e.g., Williams and Harris, 2001), but thus far, relaxation has not been linked to depleted self-control strength in sport and exercise psychology, and future research may aim to test this assumed relationship.

Another possibility to reduce the negative effects of ego depletion on sport performance is reported by Englert and Bertrams (2015b; see also Graham et al., 2014a). They manipulated the level of perceived autonomy while working on a primary self-control task because it was the participants’ decision to work on the task or to not work on the task. In a next step, the participants completed a series of tennis serves under pressure (cf., Guillot et al., 2013). Results revealed that participants that felt a higher level of autonomy while working on the primary self-control task performed significantly better in the subsequent tennis serve task. These results are in line with Muraven (2008), who demonstrated that higher levels of perceived autonomy are less depleting than are externally enforced self-control acts, and are thus beneficial for subsequent self-control performance. The explanation given by Muraven (2008) is that if participants have the feeling that it is their decision to work on a self-control task, they are less reluctant to do so, and do not need to override aversive impulses. In sports, it also has been demonstrated that a more autonomy-supportive coaching style is associated with higher motivation, and possibly better performance (e.g., Goudas et al., 1995). Taken together, it may be beneficial as a coach to grant the athletes higher levels of autonomy.

Finally, implementation intentions, or so-called if–then plans, also seem to be powerful tools to prevent ego depletion effects (e.g., Webb and Sheeran, 2003). According to Gollwitzer (1999), an implementation intention requires an individual to create specific intentions and to precisely state when to execute the intention (e.g., “When I am coming home from work, I will go to the gym”). This leads to an association between a specific situation and the behavior that needs to be performed in the given situation. When the specified situation occurs the planned behavior then gets *automatically* activated. As proposed by Gollwitzer (1999), automatic behavior does not require self-control strength meaning that it should not suffer from a temporary depletion of self-control strength. Implementation intentions are already frequently used in sport and exercise (e.g., Hagger and Luszczynska, 2014), and future research should try to investigate the specific relations between implementation intentions and ego depletion in this context.

## General Discussion

The strength model of self-control (e.g., Baumeister et al., 1994) offers potential explanations of why athletes sometimes choke under pressure (e.g., Englert and Bertrams, 2012), can be less persistent in straining physical exercises (e.g., Bray et al., 2008), have difficulties regulating their impulses (e.g., McEwan et al., 2013), or do not always adhere to their exercise routines (e.g., Martin Ginis and Bray, 2010). According to Baumeister and Colleagues (1994), all self-control acts are based on one global resource, which can become temporarily depleted after a primary self-control act. In a state of ego depletion, dominant impulses break through and—as a consequence—subsequent self-control performance may suffer (e.g., Govorun and Payne, 2006). The following discussion aims to address five important questions: (1) Is ego depletion actually relevant in sport and exercise? (2) Are there any unintended effects of ego depletion tasks on other psychological processes? (3) Are there alternative explanations for the ego depletion effect? (4) What is the difference between ego depletion and mental fatigue? (5) Is there a publication bias in ego depletion research?

The first question regards the tasks designed to manipulate self-control strength in ego depletion research. In most experiments reviewed thus far, the Stroop task (e.g., Bray et al., 2008), an emotion suppression task (e.g., Wagstaff, 2014), or the transcription task (e.g., Bertrams et al., 2010) were used for that cause. The transcription task asks participants to transcribe a neutral text onto a separate sheet of paper. Participants from a control condition are asked to transcribe the text conventionally, which does not require self-control strength, while participants in the depletion condition are instructed to omit specific letters while transcribing the text because overwriting dominant writing tendencies requires self-control strength. Although these tasks have been found to be reliable (e.g., Englert and Bertrams, 2012; Furley et al., 2013) and even though the effect of ego depletion is not domain specific (e.g., Muraven et al., 1998), the tasks are not sport-related, which raises the question of whether ego depletion is actually relevant in sport and exercise. There are two studies that tried to apply more sport-related ego depletion manipulation tasks. Englert and Bertrams (2014b) vicariously depleted their participants by asking them to read a story about a soccer player who had to regulate himself during the whole duration of a soccer match. After having read the story, the participants were asked to take the perspective of the described soccer player. When picturing themselves in the shoes of the depleted athlete described in the story, participants performed significantly worse in a subsequent self-control task. In another study, Gröpel et al. (2014) asked their participants to partake in a straining, rigorous workout program for 15 min which also lead to a temporary depletion of self-control strength. To make a stronger case for the relevance of ego depletion in sports, more sports-related ego depletion manipulation tasks should be developed.

The second question regards the possibility of unintended effects of ego depletion tasks on other psychological processes. In most studies on ego depletion, participants from the depletion condition have to perform primary tasks that are more difficult than the primary tasks used in the control conditions (e.g., the Stroop task; e.g., Bray et al., 2008). Therefore, these differences in difficulty may negatively affect other unintended psychological aspects apart from self-control strength. Some alternative explanations have been ruled out by previous studies, indicating that the negative effects of ego depletion on performance were not caused by differences in perceived self-efficacy (e.g., Wallace and Baumeister, 2002), mood differences (e.g., Baumeister et al., 1998; Muraven et al., 1998), or differences in motivation (e.g., Muraven et al., 2008; Graham et al., 2014a). Nonetheless, it seems highly important to control for unintended effects of the ego depletion manipulation tasks on other psychological processes.

The third question regards potential alternative explanations of the ego depletion effect. Recently, the assumptions of the strength model of self-control have been criticized by several researchers, who argue that impaired self-control performance after a primary self-control task is not caused by a temporary exhaustion of a limited resource. It is argued that ego depletion instead is caused by motivational shifts (e.g., Inzlicht and Schmeichel, 2012), resource allocation (Beedie and Lane, 2012), or subjective implicit theories about willpower (Job et al., 2010). These alternative approaches do not neglect the assumption that there is actually a negative carry-over effect of prior acts of self-control on subsequent performance, and the studies reviewed above, as well as the strong effect sizes in the meta-analysis by Hagger et al. (2010b), also deliver empirical support for this assumption. Nonetheless, future research should try to investigate further into the actual processes behind the ego depletion effect.

The fourth question regards the difference between mental fatigue and ego depletion. Marcora et al. (2009) define mental fatigue as a psychobiological state which is caused by prolonged periods of demanding cognitive activity, which at first sight seems to be equivalent to the definition of ego depletion proposed by Baumeister and Colleagues (1998). Hagger et al. (2010a, p. 67) in their review article came to the conclusion that “mental fatigue is therefore an analog for ego depletion and likely coincides with the depletion of self-control”. However, the results are mixed, as some studies reported higher levels of mental fatigue in depleted individuals as compared to non-depleted individuals (e.g., Bray et al., 2011) while other studies did not find any significant differences in self-reported mental fatigue after finishing an ego depletion task (e.g., Bray et al., 2008, 2012; Vohs et al., 2011). There seems to be a crucial difference between these two psychological constructs: Tasks being used to induce mental fatigue usually last significantly longer than the tasks applied in ego depletion research, as for instance Marcora et al. (2009) asked their participants to work on a cognitively demanding task for 90 min. On the contrary, in ego depletion research the tasks designed to deplete self-control strength usually do not last as long. For instance, in a study by Bray et al. (2015) participants in the depletion condition worked on a depleting Stroop task that lasted only 5 min. In regard to the studies by Bray et al. (2008, 2012), Pageaux et al. (2013, p. 2255) conclude that “in these studies, mental exertion was not prolonged enough to induce subjective feelings of mental fatigue”. Future research should try to continue to get a better understanding of the commonalities and the differences between ego depletion and mental fatigue.

Finally, a recent meta-analysis by Carter et al. (2015) came to the conclusion that the ego depletion effect may have been overestimated in previous research and that a publication bias may have led to the strong effect sizes reported in the meta-analysis by Hagger et al. (2010b). The studies from the field of sport and exercise psychology presented in the present paper are more in line with the meta-analysis conducted by Hagger et al. (2010b). However, it seems highly necessary to dig deeper into the inconsistent results reported in these two meta-analyses to make a stronger case for the ego depletion effect.

This paper aimed to give an overview of recent advances on ego depletion in sport and exercise psychology. The reviewed manuscripts demonstrate that self-control strength seems to be an important psychological variable that requires more research. Interventions aiming to improve and restore self-control strength need to be developed and tested to encourage athletes to perform at their highest levels.

## Author Contributions

CE wrote the manuscript and approves the final version of the manuscript. The author agrees to be accountable for all aspects of the work in ensuring that questions related to the accuracy or integrity of any part of the work are appropriately investigated and resolved.

## Conflict of Interest Statement

The author declares that the research was conducted in the absence of any commercial or financial relationships that could be construed as a potential conflict of interest.

The reviewer SV and handling Editor declared their shared affiliation, and the handling Editor states that the process nevertheless met the standards of a fair and objective review.
